# Nonfatal Injuries and Psychosocial Correlates among Middle School Students in Cambodia and Vietnam

**DOI:** 10.3390/ijerph14030280

**Published:** 2017-03-08

**Authors:** Karl Peltzer, Supa Pengpid

**Affiliations:** 1 ASEAN Institute for Health Development, Mahidol University, Salaya, Phutthamonthon, Nakhon Pathom 73170, Thailand; supaprom@yahoo.com; 2 Department of Research & Innovation, University of Limpopo, Sovenga 0727, South Africa; 3 HIV/AIDS/STIs and TB (HAST), Human Sciences Research Council, Pretoria 0001, South Africa

**Keywords:** injury, substance use, psychosocial factors, middle school children, Cambodia, Vietnam

## Abstract

The aim of the study was to estimate the prevalence and psychosocial correlates of nonfatal injury among middle school students in Cambodia and Vietnam. Cross-sectional data from 7137 school children (mean age 15.5 years, SD = 1.4) who were randomly sampled for participation in nationally representative Global School-based Health Surveys (GSHS) in Cambodia and Vietnam were analyzed. The proportion of school children reporting one or more serious injuries in the past year was 22.6% among boys and 17.5% among girls in Cambodia and 34.3% among boys and 25.1% among girls in Vietnam. The most prevalent cause of the most serious injury in Cambodia was traffic injuries (4.7% among boys and 4.3% among girls) and in Vietnam it was falls (10.0% among boys and 7.0% among girls). In multinomial logistic regression analyses, experiencing hunger (as an indicator for low socioeconomic status) and drug use were associated with having sustained one injury and two or more injuries in the past 12 months in Cambodia. In addition, poor mental health was associated with two or more injuries. In Vietnam, being male, experiencing hunger, current alcohol use, poor mental health and ever having had sex were associated with having sustained one injury and two or more injuries in the past 12 months. Several psychosocial variables were identified which could help in designing injury prevention strategies among middle school children in Cambodia and Vietnam.

## 1. Introduction

Injuries among adolescents are a major cause of disability and death in high-income [1] and low- and middle-income countries alike, including countries in Southeast Asia [2,3], constituting a huge public health problem [4]. Investigating injury prevalence and its risk factors can provide important information for injury prevention strategies [5]. There is a lack of recent data on the national injury prevalence and its psychosocial correlates among adolescents in Cambodia and Vietnam.

The prevalence of having sustained one or more serious injuries in the past year among adolescents in Southeast Asian countries was 45.9% in Indonesia [6], 34.9% in Malaysia [7], 27.0% in Myanmar [6], 46.8% in Thailand [6], and globally (25 developing countries) 44% among boys and 30% among girls [8]. In a large national Cambodia Accident and Injury Survey (CAIS) in 2007, the past year injury morbidity prevalence was 5.3% among 15- to 17-year-old boys and 2.7% among girls, and the leading causes of injury morbidity in this 15- to 17-year-old age group were road traffic, followed by sharp objects, animal injuries, falls, violence, falling objects, blunt objects and burns [9]. In a large study among adolescents (15–19 years) in Vietnam in 2003, Linnan et al. [10] found an annual injury prevalence of 5%. Falls were the main cause of nonfatal injuries among children and adolescents (1 to 18 years), followed by animal bites, road traffic injuries, injuries from sharp objects and burns in Vietnam [10]. In another local study in Vietnam, unintentional injuries among adolescents (14–16 years) had a prevalence of 8.2% in the past three years [11], while recreation and sports injuries were at 3.1%, followed by transportation injuries (2.9%) and work injuries (2.2%) [11]. In the Survey Assessment of Vietnamese Youth (14–25 years) in 2009, 10.6% had experienced a road traffic injury in the past year [12].

Factors associated with nonfatal injuries among adolescents include, as reviewed in Peltzer and Pengpid [7], socioeconomic factors (male gender, low socioeconomic status) and psychosocial factors such as poor mental health, substance use (smoking and drinking alcohol), risk-taking behaviors and obesity. The aim of the study was to estimate the prevalence and psychosocial correlates of nonfatal injuries among middle school students in Cambodia and Vietnam using the most recent data (2013) available from the “Global School-based Health Survey” (GSHS). The research questions for this study include: (1) What is the prevalence of annual serious injuries and the prevalence of the most common types and causes of annual serious injuries among boys and girls attending middle schools in Cambodia and Vietnam?; (2) What are the socioeconomic and psychosocial factors associated with the risk of one and multiple injuries in Cambodia and Vietnam? Using the available sociodemographic and psychosocial indicators in relation to injury occurrence from the GSHS data from Cambodia and Vietnam, results from this study may provide strategic information for adolescent injury prevention.

## 2. Methodology

### 2.1. Description of Survey and Study Population

Developed by the World Health Organization (WHO) and other United Nations agencies, with technical support from the Centers for Diseases Control and Prevention (CDC), the GSHS aims to provide data on health behaviors and protective factors among students in low- and middle-income countries around the world in order to inform youth health programs and policies [13]. This study was a secondary analysis of data from the GSHS from Cambodia and Vietnam. GSHS details and data can be accessed [13]. “The 2013 Cambodia GSHS is a school-based survey conducted primarily among students aged 13–17 years. All youth who were studying in grades 7–12 in Cambodia in school year 2012–2013 were included in the sampling frame. The 2013 Cambodia GSHS employed a two-stage cluster sample design to produce a representative sample of students: school level and class level. Fifty schools were selected to participate in this survey, 25 schools in urban and another 25 schools in rural locations” [13]. “The 2013 Vietnam GSHS was a school-based survey of students in grades 8–12, which are typically attended by students aged 13–17 years. A three-stage cluster sampling design was used to produce data representative of students in grades 8–12. At the first stage, provinces were selected with probability proportional to enrollment size. At the second stage, schools were selected with probability proportional to enrollment size. At the third stage, classes were randomly selected and all students in selected classes were eligible to participate” [13].

Students completed a self-administered questionnaire under the supervision of trained research assistants during classroom periods. The secondary school gross enrolment ratio (GER) was 45% in 2008 in Cambodia and 77% in 2010 in Vietnam [14]; GER is the total enrolment within a country “in a specific level of education, regardless of age, expressed as a percentage of the population in the official age group corresponding to this level of education” [14]. The study protocol was approved by National Ethics Committees, and informed consent was obtained from the students, parents and/or school officials.

### 2.2. Measures

The GSHS Cambodia and Vietnam questionnaires were used in this investigation; they contain modules on tobacco, alcohol, and other drug use; unintentional injuries and violence, a range of other health related behaviors, and demographics [13]. The study variables were selected based on previous studies [5,6,7] and availability from the GSHS Cambodia and Vietnam [13]; they are described in Table 1. In addition, body weight and height were assessed by self-report. International age- and sex-specific child body mass index (BMI) were used and calculated as weight/height^2^ (kg/m^2^), cut-points were used to define overweight and obesity [15]. Participants were categorized as overweight or obese if their Body Mass Index (BMI) was ”>+1 SD and >+2 SD respectively from the median for BMI for age and sex” [15]. Good reliability of the GSHS questionnaire was found in a test-retest reliability study: “Average agreement between test and retest was 77%, and average Cohen’s kappa was 0.47” [16].

### 2.3. Data Analysis

Data analysis was conducted using STATA software version 13.0 (Stata Corporation, College Station, TX, USA). This software provides robust standard errors that account for the cluster sampling design. Multinominal logistic regression analyses were performed to obtain relative risk ratios and 95% confidence intervals (CI) to estimate the associations between sociodemographic, substance use, mental health, sexual behavior, BMI weight status with one injury and two or more injuries in the past 12 months, for Cambodia and Vietnam separately. Independent variables which significantly increased the injury risk in univariate analysis were included in the multivariable model. *p* < 0.05 was considered significant. The reported 95% confidence intervals and the *p*-values are both adjusted for the multi-stage stratified cluster sample design of the survey.

## 3. Results

### 3.1. Sample Characteristics

The total study sample included 7137 middle school children (3806 from Cambodia and 3331 from Vietnam), with a mean age of 15.5 years (SD = 1.4), and 46.5% were male and 53.3% were female. The year of the GSHS was 2013, and the overall response rate was 85% (100% school response rate and 85% student response rate) in Cambodia and 96% (100% school response rate and 96% student response rate) in Vietnam. The samples in terms of age, sex and educational characteristics are described in Table 2.

### 3.2. Descriptive Results

The percentage of middle school children with one or more serious injuries in the past year was 22.6% among boys and 17.5% among girls in Cambodia and 34.3% among boys and 25.1% among girls in Vietnam. The major causes of the most serious injury were “motor vehicle accident or hit by a motor vehicle” (4.7% among boys and 4.3% among girls) in Cambodia, followed by “fall” (4.3% and 2.9% among boys and girls, respectively) and “something fell on me or hit me” (0.8% and 0.9% among boys and girls, respectively). In Vietnam, the major causes of the most serious injury were “fall” (10.0% among boys and 7.0% among girls), followed by “motor vehicle accident or hit by a motor vehicle” (3.4% among boys and 4.1% among girls), and “was attacked or abused or was fighting with someone” (2.2% among boys and 0.4% among girls). The most frequent type of injury sustained was “a broken bone or dislocated joint” in Cambodia (3.6% among boys) and in Vietnam (7.0% among boys and 3.1% among girls), while the most common type of injury among girls in Cambodia was a concussion (3.9%). In addition, 15.8% of boys and 13.4% of girls in Vietnam reported other types of injuries (see Table 3).

### 3.3. Associations with Injury Prevalence

In multinomial logistic regression analyses, experience of hunger as an indicator for low socioeconomic status and drug use were associated with one injury and two or more injuries in the past 12 months in Cambodia. In addition, poor mental health (suicidal ideation and loneliness) was associated with two or more injuries in the past 12 months in Cambodia. Confounding variables, identified from previous studies [5,6,7,8,17] (having been attacked, having been in a physical fight, and having been bullied in past month) and showing a strong interaction with the injury outcome in this analysis, were highly associated with one injury and two or more injuries in the past 12 months in Cambodia (see Table 4) in bivariate analysis.

In multinomial logistic regression analyses, being male, experiencing hunger as an indicator for low socioeconomic status, current alcohol use, poor mental health (suicidal ideation and loneliness) and ever having had sex was associated with one injury and two or more injuries in the past 12 months in Vietnam. In addition, older children (17–18 years or older) were less likely to report two or more injuries in the past 12 months than younger children in Vietnam. Confounding variables (having been attacked, having been in a physical fight, and having been bullied in past month) were highly associated with one injury and two or more injuries in the past 12 months in Vietnam (see Table 5) in bivariate analysis.

## 4. Discussion

The study investigated, in large, nationally representative samples of middle school students in Cambodia and Vietnam, the prevalence and correlates of nonfatal injury occurrence. The annual nonfatal injury prevalence found was higher in Vietnam (34.3% among boys and 25.1% among girls) than in Cambodia (22.6% among boys and 17.5% among girls), which seems lower than the median from global studies of 25 developing countries (44% among boys and 30% among girls) [8] and 47 developing countries (median 40%) [18], but similar to findings from Myanmar (27%) [6]. However, the current annual prevalence of nonfatal injury in Cambodia and Vietnam seems to be higher than in previous large, population-based surveys among adolescents in Cambodia (5.3% among males and 2.7% among females) [9] and Vietnam (5%) [10].

Among the different causes of injury, the study found that the highest annual prevalence rate was traffic-related injuries in Cambodia and falls in Vietnam, which was similar to findings from various studies in Southeast Asian countries [6,7,9,10]. Motor vehicle or transport-related injuries may be related to poor traffic infrastructure, including road conditions, poor public transport, pedestrian injuries, lack of helmet use, non-use of seatbelts, and alcohol [11]. In the previous large child injury survey in Vietnam, the most prevalent non-fatal injury type was also falls [10]. In the same survey in Vietnam, 60.2% of falls were caused by slipping, followed by jumping, being hit, epileptic seizures and overbalancing [10]. The most frequent type of injury sustained in this study was “a broken bone or dislocated joint” and “a concussion or other head or neck injury”, while in previous surveys in the region “a broken bone or dislocated joint” was also the most prevalent injury type, followed by “a cut, puncture, or stab wound” and a concussion in third place [6,7].

In concurrence with previous studies [6,7,11,19], male gender was, in this study, in Vietnam but not in Cambodia, associated with injury. Further, experiencing hunger as an indicator of low socioeconomic status was, in this survey, associated with annual injury prevalence, as also found in previous studies [6,7,11]. It is possible that adolescents coming from a lower socioeconomic status may be more exposed to work-related injuries [11], and experience greater material deprivation and societal obstacles to protect them from injury [20].

Moreover, poor mental health was significantly associated with injury in this study, in particular with having sustained two or more injuries in the past 12 months. This result was also previously found [6,7,17,18]. Substance use (ever using drugs in Cambodia and current alcohol use in Vietnam) was, as also identified in previous studies [6,17], associated with injury in this adolescent population. A possible reason for this association between substance use and injury may be a lack of parental supervision which may substance use and injury due to exposure to hazardous environments more likely to occur [21]. The GSHS conducted in Cambodia and Vietnam did not include information on protective factors such as parental supervision, and it is not known if substance use was initiated prior to the occurrence of or following the injury. In addition, one health risk behavior (ever having had sex) was associated with injury in Vietnam and in bivariate analysis in Cambodia, as also found in a previous study in Malaysia [7]. Further, aggressive behavior (having been attacked, having been bullied and having been in a physical fight) was highly associated with injury prevalence. It is possible that substance use may increase aggressive behavior and also the risks of injuries through, for example, falls and traffic-related trauma [22]. Poor mental health, substance use and other health risk behaviors seem to play a role in adolescent injuries, which calls for strategies to include psycho-behavioral risk reduction in adolescent injury prevention programs [23]. Contrary to a previous investigation [24], being overweight or obesity was not related to injury prevalence in this study.

The high frequency and burden of injuries on morbidity and mortality, together with the high success potential of prevention strategies such as training and education and the use of safety equipment, make injury prevention a public health priority to improve adolescent health in the future [1,25]. There is an urgent need for intervention programs in schools that address the burden of injuries to adolescents, and the need for schools to address injury prevention as a priority educational need in these two countries (to be included in the curriculum).

### Study Limitations

The study sample focused on school-going adolescents and findings cannot be generalized to all adolescents in Cambodia and Vietnam. Moreover, the study information was assessed by self-report, including height and weight, which may have introduced a reporting bias. Further, as this was a cross-sectional survey, no causative conclusions may be drawn. School students were surveyed on a particular day and the students could have been absent for injuries. The analysis was restricted to the study variables that had been included in the GSHS in Cambodia and Vietnam.

## 5. Conclusions

The study found, in two nationally representative samples of school-going adolescents in Cambodia and Vietnam, that a significant proportion had sustained one or more injuries in the past year. Lower socioeconomic status, poor mental health, substance use and other health risk behaviors were identified as possible risk factors for injuries that can help guide injury prevention programming in this adolescent population.

## Figures and Tables

**Table 1:** Variable description.

Variables	Question	Response Options
Injury	“During the past 12 months, how many times were you seriously injured? (An injury is serious when it makes you miss at least one full day of usual activities (such as school, sports, or a job) or requires treatment by a doctor or medical personnel.)”	1 = 0 times, 2 = 1, 3 = 2–3, 4 = 4–5, 5 = 6–7, 6 = 8–9, 7 = 10–11, 8 = 12 or more times
	“During the past 12 months, what was the most serious injury that happened to you?”	1 = I was not seriously injured during the past 12 months; 2 = I had a broken bone or a dislocated joint; etc. see Table 2
	“During the past 12 months, what was the major cause of the most serious injury that happened to you?”	1 = I was not seriously injured during the past 12 months; 2 = I was in a motor vehicle accident or hit by a motor vehicle etc. see Table 2
Current smoking cigarettes	“During the past 30 days, on how many days did you smoke cigarettes?”	1 = 0 days to 7 = All 30 days
Current other tobacco use	“During the past 30 days, on how many days did you use tobacco products other than cigarettes such as chewing tobacco with or without betel quits or cigars in Cambodia and thuoc lao in Vietnam?”	1 = 0 days to 7 = All 30 days
Current alcohol use	“During the past 30 days, on how many days did you have at least one drink containing alcohol?”	1 = 0 days to 7 = All 30 days
Lifetime drug use	“How old were you when you first used drugs?”	1 = never to 8 = 18 years old or older
Anxiety	“During the past 12 months, how often have you been so worried about something that you could not sleep at night?”	1 = never to 5 = always
Suicidal ideation	“During the past 12 months, did you ever seriously consider attempting suicide?”	1 = yes, 2 = no
Loneliness	“During the past 12 months, how often have you felt lonely?”	1 = never to 5 = always
No close friends	“How many close friends do you have?”	1 = 0 to 4 = 3 or more
Ever sex	“Have you ever had sexual intercourse?”	1 = yes, 2 = no
Attacked	“During the past 12 months, how many times were you physically attacked?”	1 = 0 times to 8 = 12 or more times
In a physical fight	“During the past 12 months, how many times were you in a physical fight?”	1 = 0 times to 8 = 12 or more times
Bullied	“During the past 30 days, on how many days were you bullied?”	1 = 0 days to 7 = All 30 days

**Table 2:** Sample characteristics.

Variable	Cambodia		Vietnam	
	*n* = 3806 (Unweighted Frequency)	% (Weighted Percent) (95% CI)	*n* = 3331 (Unweighted Frequency)	% (Weighted Percent) (95% CI)
Sex				
Male	1791	47.6 (45.2, 50.0)	1557	46.7 (44.5, 48.9)
Female	2993	52.4 (50.0, 54.8)	1765	53.3 (51.1, 55.5)
Age				
13–14 years or younger	1184	32.9 (27.7, 38.6)	896	21.5 (16.1, 28.2)
15–16 years	1161	33.5 (28.1, 39.4)	1397	42.2 (35.7, 49.0)
17 years or older	1453	33.5 (27.7, 40.0)	1032	36.2 (29.7, 43.3)
School grade				
Grade 7	893	25.7 (18.1, 35.1)	1	0.0
Grade 8	685	20.2 (14.1, 28.2)	965	23.2 (17.0, 30.2)
Grade 9	2210	54.0 (45.4, 62.5)	2347	76.8 (69.8, 82.6)

**Table 3:** Annual prevalence of injury events, cause and type of injury by gender in Cambodia and Vietnam.

Variable	Cambodia		Vietnam	
	Boys	Girls	Boys	Girls
	*n* (%)	*n* (%)	*n* (%)	*n* (%)
Injury (in the past 12 months)				
Injured once	236 (15.2)	224 (12.1)	247 (18.8)	226 (15.4)
Injured more than once	121 (7.4)	94 (5.4)	196 (15.5)	147 (9.7)
Cause (of most serious injury)				
Traffic injury	72 (4.7)	93 (4.3)	42 (3.4)	53 (4.1)
Fall	63 (4.3)	44 (2.9)	133 (10.0)	108 (7.0)
Struck/hit by object	15 (0.8)	12 (0.9)	11 (0.8)	12 (0.9)
Struck/hit by person	7 (0.3)	3 (0.3)	27 (2.2)	6 (0.4)
Fire, flames or heat	6 (0.4)	10 (0.6)	2 (0.1)	9 (0.5)
Poisoning	10 (0.6)	16 (0.6)	0 (0.0)	2 (0.1)
Other	57 (3.3)	47 (2.6)	158 (12.4)	114 (7.5)
Type of injury (of most serious injury)				
Fracture or dislocation	66 (3.6)	30 (1.8)	93 (7.0)	44 (3.1)
Cuts, bites or open wound	14 (1.1)	8 (0.5)	13 (1.0)	2 (0.1)
Concussion	54 (3.4)	77 (3.9)	23 (1.8)	11 (0.9)
Gunshot wound	2 (0.1)	3 (0.2)	3 (0.2)	3 (0.1)
Burns	11 (0.9)	9 (0.6)	6 (0.4)	13 (0.8)
Poisoned	9 (0.4)	12 (0.5)	2 (0.1)	1 (0.1)
Other	60 (4.0)	67 (3.3)	215 (15.8)	207 (13.4)

**Table 4:** Relative risk ratios and 95% confidence intervals from a multinomial logistic regression model predicting one injury and two or more injuries in Cambodia.

Variables	One Injury		Two or More Injuries	
	RRR (95% CI)	RRR (95% CI)	RRR (95% CI)	RRR (95% CI)
	Crude	Adjusted	Crude	Adjusted
Age in years				
13–14 or younger (33.3%)	1 (Reference)	1 (Reference)	1 (Reference)	1 (Reference)
15–16 (33.4%)	1.12 (0.76, 1.64)	1.34 (0.94, 1.91)	1.21 (0.88, 165)	1.38 (0.89, 2.15)
17–18 or older (33.6%)	1.06 (0.81, 1.36)	1.23 (0.78, 1.96)	1.49 (1.05, 2.11) *	1.37 (0.87, 2.15)
Gender				
Female (52.3%)	1 (Reference)	1 (Reference)	1 (Reference)	1 (Reference)
Male (47.7%)	1.34 (0.87, 2.06)	1.14 (0.70, 1.86)	1.44 (1.18, 1.79) **	1.15 (0.86, 1.54)
Hunger				
Never (45.0%)	1 (Reference)	1 (Reference)	1 (Reference)	1 (Reference)
Rarely (19.7%)	1.09 (0.71, 1.69)	0.97 (0.61, 1.56)	1.97 (1.19, 3.23) **	1.70 (1.06, 2.72) *
Sometime/mostly/always (35.3%)	2.39 (1.78, 3.63) ***	2.21 (1.51, 3.23) ***	3.40 (2.20, 5.26) ***	3.18 (1.81, 5.58) ***
Current any tobacco use (3.6%)	5.12 (3.14, 8.35) ***	2.04 (0.97, 4.31)	6.27 (2.61, 15.02) ***	1.06 (0.46, 2.43)
Current drinking (10.0%)	2.13 (1.30, 3.50) **	1.66 (0.89, 3.10)	2.87 (1.63, 5.05) ***	1.23 (0.60, 2.51)
Ever drug use (3.5%)	3.66 (2.09, 6.44) ***	2.01 (1.14, 3.56) *	6.25 (3.17, 12.33) ***	3.91 (1.82, 8.41) ***
Anxiety (6.0%)	1.64 (1.02, 2.65) *	1.01 (0.46, 1.78)	3.72 (2.19, 6.33) ***	2.23 (1.25, 4.00) **
Suicidal ideation (6.4%)	1.60 (0.83, 3.07)	0.99 (0.45, 2.15)	2.96 (1.97, 4.45) ***	1.33 (0.81, 2.19)
Loneliness (5.7%)	1.57 (0.97, 2.54)	1.26 (0.69–2.32)	3.91 (2.79, 5.49) ***	1.79 (1.12, 2.85) *
No close friends (5.0%)	1.53 (0.90, 2.61)	-	1.50 (0.75, 2.99)	-
Ever sex (11.9%)	1.90 (1.39, 2.61) ***	1.33 (0.87, 2.04)	1.96 (1.30, 2.97) **	1.39 (0.91, 2.11)
Overweight or obesity (3.4%)	0.76 (0.42, 1.37)	-	0.92 (0.30, 2.83)	-
Confounding variables ^1^				
Attacked (17.5%)	3.75 (3.09, 4.56) ***		7.00 (5.00, 9.63) ***	
In a physical fight (10.5%)	3.34 (2.35, 4.74) ***		5.11 (3.37, 7.75) ***	
Bullied in the past month (22.3%)	3.13 (2.41, 4.06 ***		7.83 (4.61, 13.31) ***	

RRR = Relative Risk Ratio; CI = Confidence Interval; *** *p* < 0.000, ** *p* < 0.01, * *p* < 0.05; ^1^ Not included in the adjusted analysis.

**Table 5:** Relative risk ratios and 95% confidence intervals from a multinomial logistic regression model predicting one injury and two or more with injuries in Vietnam.

Variables	One Injury		Two or More Injuries	
	RRR (95% CI)	RRR (95% CI)	RRR (95% CI)	RRR (95% CI)
	Crude	Adjusted	Crude	Adjusted
Age in years				
13–14 or younger (21.7%)	1 (Reference)	1 (Reference)	1 (Reference)	1 (Reference)
15–16 (42.1%)	1.03 (0.76, 1.39)	0.93 (0.66, 1.30)	0.83 (0.58, 1.20)	0.78 (0.52, 1.16)
17–18 or older (36.3%)	0.88 (0.59, 1.32)	0.70 (0.48, 1.02)	0.70 (0.51, 0.96) *	0.56 (0.40, 0.78) ***
Gender				
Female (53.1%)	1 (Reference)	1 (Reference)	1 (Reference)	1 (Reference)
Male (46.9%)	1.39 (1.16, 1.66) ***	1.47 (1.17, 1.84) **	1.83 (1.40, 2.37) ***	1.90 (1.47, 2.44) ***
Hunger				
Never (47.6%)	1 (Reference)	1 (Reference)	1 (Reference)	1 (Reference)
Rarely (29.6%)	1.48 (1.13, 1.93) **	1.42 (1.10, 1.84) **	1.55 (1.08, 2.21) *	1.21 (0.84, 1.74)
Sometime/mostly/always (22.8%)	1.92 (1.49, 2.48) ***	1.80 (1.32, 2.45) ***	2.09 (1.53, 2.87) ***	1.93 (1.30, 2.86) **
Current any tobacco use (5.2%)	1.75 (1.05, 2.90) *	0.95 (0.54, 1.67)	2.85 (1.50, 5.41) **	1.29 (0.63, 2.67)
Current drinking (24.9%)	1.74 (1.30, 2.33) ***	1.53 (1.08, 2.17) *	2.13 (1.64, 2.77) ***	1.56 (1.19, 2.04) **
Ever drug use (1.4%)	1.27 (0.29, 5.48)	1.21 (0.03, 2.68)	5.32 (2.18, 12.96) ***	1.57 (0.41, 5.98)
Suicidal ideation (16.9%)	2.27 (1.77, 2.92) ***	1.95 (1.44, 2.65) ***	2.29 (1.66, 3.16) ***	1.92 (1.26, 2.91) **
Loneliness (11.5%)	1.76 (1.31, 2.37) ***	1.47 (1.05, 2.06) *	2.62 (1.97, 3.47) ***	2.01 (1.47, 2.74) ***
No close friends (5.5%)	1.47 (0.90, 2.39)	-	1.23 (0.79, 1.91)	-
Ever sex (6.5%)	2.14 (1.06, 4.37) *	1.83 (1.01, 3.32) *	2.78 (1.70, 4.56) ***	1.90 (1.13, 3.19) *
Overweight or obesity (5.8%)	0.92 (0.55, 1.56)	-	1.42 (0.95, 2.12)	-
Confounding variables ^1^				
Attacked (21.0%)	2.43 (1.98, 3.00) ***		3.92 (2.96, 5.20) ***	
In a physical fight (16.6%)	2.74 (2.13, 3.52) ***		4.21 (3.08, 5.76) ***	
Bullied in the past month (22.7%)	2.73 (2.15, 3.45)***		3.72 (2.80, 4.94) ***	

RRR = Relative Risk Ratio; CI = Confidence Interval; *** *p* < 0.000, ** *p* < 0.01, * *p* < 0.05; ^1^ Not included in the adjusted analysis.
